# Synergistic effect of nano-potassium and chitosan as stimulants inducing growth and yield of bird of paradise (*Sterlitiza reginae* L.) in newly lands

**DOI:** 10.1080/19420889.2024.2406754

**Published:** 2024-09-26

**Authors:** Eman A. Swedan, Kholoud Shible, Yassin M. Yassin, Aleksandra Glowacka, Mohamed A. A. Ahmed

**Affiliations:** a Horticulture Department, Faculty of Agriculture, Damanhour University, Buhaura, Egypt; b Department of Horticulture, Faculty of Agriculture, Sohag University, Sohag, Egypt; c Department of Plant Cultivation Technology and Commodity Sciences, University of Life Sciences in Lublin, Lublin, Poland; d Plant Production Department (Horticulture - Medicinal and Aromatic Plants), Faculty of Agriculture (Saba Basha), Alexandria University, Alexandria, Egypt; e National Key Laboratory for Tropical Crop Breeding/Coconut Research Institute, Chinese Academy of Tropical Agricultural Sciences, Hainan, China

**Keywords:** Bird of paradise, chitosan, growth and yield characters, nano-potassium, *Sterlitiza reginae*

## Abstract

The bird of paradise plant is a clumping tropical species native to South Africa. It is a dramatic plant with distinctive iridescent orange and midnight blue flowers that resemble an exotic bird peeking out from the broad leaves in autumn, winter and spring. An experiment was conducted during the two seasons of 2021 and 2022 at a private farm in Damanhour, Beheira Governorate, Egypt (31“°” 04 ”°“N, 30“°” 47’ °E) to study the effect different concentrations of nano-potassium and chitosan and their combinations on the bird of Paradise (*Sterlitiza reginae*). The experiment was conducted in a randomized complete block in a split-plot design with five replicates; nano-potassium was used at 0, 100, 150, and 200 mg/l and assigned to the main plots, whereas the sub-plots involved 0, 0.25, 0.50 and 0.75 g/l of chitosan. An increase in plant height and leaf length was recorded when the plants were treated with 200 mg/l nano-potassium and 0.75 g/l chitosan. Spraying plants with concentrations of 150 mg/l nano-potassium and 0.75 g/l chitosan is associated with the superiority of *S. reginae* plants in other traits, such as leaves wide, number of leaves/plant, days to flowering, number of inflorescence/plant, number of florets/inflorescence, stalk length and diameter, inflorescence weight, longevity of inflorescence, and flowering period, compared to the other treatments. We conclude that adding nano-potassium and/or chitosan to the bird of paradise plant leads to an improvement in terms of vegetative and yield characteristics under newly reclaimed lands.

## Introduction

The bird of paradise plant (*Strelitzia reginae* Ait.) is an evergreen perennial herbaceous species belonging to the family Strelitziaceae, cultivated in the regions of moderate subtropical climate. The brilliant colors and unusual appearance of the flowers have made it exceptionally popular as cut flower. Therefore, the plant is grown in many parts of the world for producing cut flowers for the domestic and international markets. It is a very popular flowering plant that grows up to 90 cm in height, with a leaf stalk length of about 45 cm and the leaf blade of the same length. Paradise plants have attractive and majestic flowers that can be produced in various subtropical and tropical regions. Its flowers are considered one of the flowers with popular markets locally and for export. It is vegetatively propagated by division of naturally developed branches known as fans. Vegetative propagation by division is limited by a low rate of multiplication being 0.5–1.5 divisions per branch per year. Branching originates in the division of the apical dome with an absolute absence of branching from axillary buds [1].

Soil type is strongly affected by the growth and productivity of these plant species. However, in Egypt, sandy soils lacking nutrients represent a large sector of reclaimed land. Therefore, it is important to provide nutrients and fertilizers to improve the productivity of this type of plant. Plant nutrition has a direct effect on the crop physiology because it plays a vital role in the productivity of any crop. However, potassium is one of the limiting elements affecting plant output in growing media. Potassium is one of the most important elements that plays a crucial role in water balance in plants. It also improves shelf life and crop quality and reduces drought-related losses [2]. In addition, providing the plant with a balanced potassium diet ensures that it is healthy, strong, and resistant to diseases. It plays a crucial role in various physiological processes such as osmoregulation, protein synthesis, cation–anion balance, and enzyme activation of enzymes [3]. Potassium, as a plant nutrient, plays an important role in controlling cell water content, biosynthesis of carbohydrates, mobilization in plant tissues, growth of meristematic tissue, and preservation of cell turgor pressure; consequently, it is important for flowering and yield of plants [4–6]. The application of potassium is especially crucial when high rates of N and P are used and when high productivity is expected [7]. Accordingly, there is a need to improve the use of potassium to maximize its use. Nano-fertilizer (NF) is regarded as an active and environmentally friendly nutrient [8,9]. Nano-fertilizer suggests new plant management strategies. Although potassium is difficult to incorporate into organic materials, it helps to improve crop growth and quality. Plant yield and quality are determined by the time of fertilization and harvesting (days after flowering) in the field. Because nanoparticles have better mobility, it can transfer nanoformulated nutrients to all parts of the plant. Nano-fertilizer outperforms even the most creative modern conventional fertilizers because of their high surface area-to-volume ratio [3]. Many studies have shown that plant height and yield increase when a nano-fertilizer is added at a recommended rate. Abdulrahman et al. [10] showed that plant height, leaf number, leaf area, chlorophyll content, dry weight, and eggplant leaf content increased when the nano-potassium fertilizer was used at a rate of 1.5 g/l. [11] showed that nano-fertilizers play a crucial role in plant nutrition, whether they are applied as soil drenches or as foliar sprays, as they work to improve the activity of the photosynthetic processes, improve the plant components of active substances, and induce the plant’s ability to withstand different stress conditions.

Chitosan is one of the most important biopolymers that stimulates plant growth characteristics, flowering behavior, and yield traits [12]. Chitosan belongs to the carbohydrate family that contains an unramified chain formula; originally formulated from the glucose circle; however, it contains a group of free amino, carbon atomnum2 (called glucose amino), which is similar to cellulose. Chemically, chitosan is a linear polymer composed of two sub-units, D-glucosamine and *N*-acetyl-D-glucosamine, linked with each other through 1,4-glycosidic bonds [13]. Chitosan can be extracted from an insect’s exoskeleton or obtained from marine crustaceans, such as crabs and prawns. Therefore, they are considered environmentally safe when used as stimulants for the growth and production of agricultural crops. In addition, it can be used to relieve various stressors when plants are exposed to drought, temperature, or salinity [14,15]. Faqir [16] described the use of chitosan in different agricultural sectors, and it is extensively applied as a fertilizer, that is, controlled-release fertilizer, liquid fertilizer, fertilizer, and micronutrient delivery to promote plant growth. A study by Parvin et al. [17] suggested that spraying tomato with chitosan alone significantly improved the growth measures, yield, and its attributes when compared with untreated plants. Sabreen et al. [18] pointed out that spraying squash crop with 0.10 g/l chitosan recorded the highest values of plant growth, productivity, and its chemical components. Chitosan-treated plants showed more leaves, shoots, flowers, and corms, as well as fewer days to flowering [19]. In the current study, we applied both nano-potassium and chitosan at different levels and their combination to study their effects on the growth, flowering, and chemical composition of the paradise plant in new lands, as well as to know the most effective levels of these materials on different traits of *Strelitzia reginae*.

## Materials and methods

### Plant materials and experimental site

The present investigation was conducted during the two seasons of 2020/2021 and 2021/2022 in a private farm in Damanhour, Beheira Governorate, Egypt (31° 04 °N, 30° 47’ °E). Eighty suckers of *Strelitzia reginae* were separated from the mother plants. Uniform-sized suckers of approximately 25 cm height were dipped in a fungicide solution and planted on February 15^th^ for both seasons in PVC pots with a diameter of 30 cm filled with sandy soil. The suckers were irrigated immediately after planting. Soil samples were obtained at a depth of 30 cm. The physical and chemical characteristics of the soil were measured according to the methods described by Jackson [20] and Black et al. [21] (Table 1). Chitosan (deacetylation degree 4–17%; molecular weight 318.53 kDa) is made from shrimp waste (*Parapenaeus longirostris*) as described by El Amerany et al. [22]. It dissolved in 0.05% (*v*/*v*) acetic acid to a final concentration of 1 g/l. From this, three other solutions of different concentrations (0.25, 0.50, and 0.75 g/l) were prepared. Distilled water served as control. Chitosan and nano-K were obtained from Al-Gomhouria Company, Assiut governorate, Egypt.

**Table 1.** Soil analysis for the physical and chemical characteristics.

Physical properties	Soil Texture		Sand %			Silt %		Clay %		
	Sandy		94.70			2.20		3.10		
Chemical properties	pH	EC (ds/m)	Soluble cations meq/L					Soluble anions meq/L		
			Na^+^	K^+^	Ca^++^		Mg^++^	CO^−3^	HCO^−3^	Cl^−^
	8.00	0.28	13.20	7.25	2.05		0.45	0.00	4.25	3.35

### Experimental design

The experiment was conducted in a randomized complete block in a split-plot design with five replicates. Nano-potassium was applied at four concentrations (0, 100, 150, and 200 mg/l) and assigned to the main plots. The sub-plots had four chitosan concentrations (0, 0.25, 0.50, and 0.75 g/l).

### The experiment treatments

The current experiments included 16 treatments, including combinations of four nano-potassium rates and four chitosan concentrations, and were replicated five times with a total of 80 pots. The plants were sprayed with different treatments at monthly intervals from mid-March to October for each season. Untreated Paradise plants were sprayed with tap water. The recommended nitrogen and phosphorus fertilizers were added to the soil as urea and super phosphate, respectively.

### Recorded data

In the 1^st^ and 2^nd^ seasons, three plants for each replication in each treatment were used to measure the plant height (cm), leaf length (cm), leaf width (cm), number of leaves/plant, days to flowering for initiation of inflorescence, number of inflorescences per plant per month, number of florets per inflorescence, stalk length (cm), stalk diameter (mm), inflorescence weight (g), longevity of inflorescence (days), and flowering period (days). Total chlorophyll (mg/g fresh weight) was determined in fresh leaves according to Dawood [23]. For N, P, and K mineral analysis, leaf samples were oven dried for 72 h at 70°C until a constant weight was reached, and then were fine ground and wet digested. N concentration was measured using the microKahl analysis method [24], and phosphorus and potassium were measured according to the method of [25].

### Statistical analysis

The recorded data in the two seasons of the experiment were collected to create an average for both seasons and subjected to analysis of variance as a simple experiment in a randomized complete block design. LSD at 5% was used to calculate the differences between means, according to ref 26.

## Results

### Plant height and leaf length

Data on the effects of nano-potassium and chitosan concentrations on the mean plant height and leaf length of *Strelitzia reginae* are shown in Table 2. As observed, nano-potassium concentrations showed significant differences in these traits. Comparative images of plant growth revealed that the foliar application of nano-potassium of 200 mg/l recorded the highest value of plant height (99.33 cm), followed by the 150 mg/l (96.33 cm) compared to the control. The longest leaves (49.67 cm) were recorded with foliar application of nano-potassium at a rate of 200 mg/l, followed by 150 mg/l (47.67 cm) when compared to untreated plants in the mean seasons. As for chitosan concentrations, it was clear that spraying *S. reginae* plants with chitosan significantly increased plant height and leaf length compared with untreated plants. Spraying chitosan at a rate of 0.75 g/l gave the tallest plants (107.50 Â cm) and longest leaves (50.92 Â cm) with a significant increase compared to untreated plants. Generally, the combination of 200 ppm nano-potassium with 0.75 g/l chitosan produced the tallest plants (112.33 cm) and longest leaves (55.00 cm) compared to the other combinations.

**Table 2.** Influence of nano-potassium and chitosan concentrations on the plant height (cm) and leaf length (cm) of *Strelitzia reginae*. The values are averages of the two experimental seasons of 2021 and 2022.

Plant height (cm)						Leaf length (cm)				
(A) Nano-K (mg/l)	(B) Chitosan (g/l)					(B) Chitosan (g/l)				
	0	0.25	0.50	0.75	M	0	0.25	0.50	0.75	M
0	74.00	86.67	95.00	102.67	89.58	40.00	43.00	46.00	47.67	44.17
50	75.33	91.33	97.00	106.00	92.42	43.00	44.00	47.00	50.00	46.00
150	78.00	95.67	102.67	109.00	96.33	44.67	46.00	49.00	51.00	47.67
200	81.00	98.00	106.00	112.33	99.33	46.00	46.67	51.00	55.00	49.67
M	77.08	92.92	100.17	107.50		43.42	44.92	48.25	50.92	
LSD 5%	A = 1.29 B = 1.03 AB = 2.19					A = 0.63 B = 0.93 AB = 1.72				

**Leaf width and number of leaves/ plants**

The leaf width and number of leaves per plant of *S. reginae* as influenced by nano-potassium and chitosan concentrations are shown in Table 3. The results revealed that these traits were considerably improved by all nano-potassium concentrations used, compared to the control. Generally, the widest leaves (16.75 cm) were from plants sprayed with 150 g/l nano-potassium, followed by 200 g/l (16.42 cm). The maximum values of the number of leaves/plant (9.06 and 9.03) were observed in plants sprayed with nano-potassium at rates of 150 and 200 mg/l, respectively. Concerning the effect of chitosan levels, it could be inferred that spraying the birds of paradise plants with growth chitosan strongly affected leaf width and the number of leaves per plant compared to untreated plants. However, the widest leaves (17.25 and 16.67 cm) were registered with plants sprayed with chitosan at the rates of 0.50 and 0.75 g/l, respectively. Meanwhile, the higher number of leaves/plant (9.72 and 8.83) was obtained from plants sprayed with 0.75 and 0.50 g/l, respectively. The interaction effect between nano-potassium and growth chitosan treatments on the leaf width and number of leaves per plant showed that the combination of 150 mg/l nano-K with chitosan 0.50 g/l resulted in the maximum leaf width (18.00 cm), while the maximum number of leaves per plant (11.30) was recorded with the combination of 150 mg/l nano-K and 0.75 g/l chitosan.

**Table 3.** Influence of nano-potassium and chitosan concentrations on the leaf width (cm) and leaf number per plant of *Strelitzia reginae*. The values are averages of the two experimental seasons of 2021 and 2022.

Leaf width (cm)						No. of leaves/plant				
(A) Nano-K (mg/l)	(B) Chitosan (g/l)					(B) Chitosan (g/l)				
	0	0.25	0.50	0.75	M	0	0.25	0.50	0.75	M
0	13.00	14.00	17.00	16.00	15.00	6.53	7.27	7.80	8.30	7.48
100	14.00	15.00	17.00	17.67	15.92	7.30	7.70	8.63	9.23	8.22
150	15.00	17.00	18.00	17.00	16.75	7.63	8.17	9.13	11.30	9.06
200	16.00	16.67	17.00	16.00	16.42	7.70	8.63	9.73	10.03	9.03
M	14.50	15.67	17.25	16.67		7.29	7.94	8.83	9.72	
LSD 5%	A = 0.79 B = 0.91 AB=1.76					A = 0.29 B = 0.21 AB = 0.46				

**Days to flowering and number of inflorescence/ plant**

The results in Table 4 show a significant effect of nano-K and chitosan concentrations as well as their interaction on the days to flowering for initiation of inflorescence and number of inflorescence/plant. The plants sprayed with 200 and 150 mg/l had the least number of days to flowering for initiation of inflorescence average at 41.26 and 41.33 days, respectively, compared with untreated plants, which gave the highest average trait value of 43.36 days. A higher number of inflorescences/plant (2.11 and 1.80) were attained due to plants sprayed with 150 and 200 mg/l, respectively. The lowest value for this trait (1.36) was recorded in the control treatment. The results in the same table indicate that spraying plants with chitosan at different levels led to a reduction in the number of days to flowering for the initiation of inflorescence and an increase in the number of inflorescences per plant/month compared to the control. Spraying plants with 0.75 g/l chitosan gave the lowest number of days to flowering and the maximum number of inflorescences per plant compared to other treatments. The results of the two traits indicated that the interaction between the concentrations of nano-potassium and chitosan was significant and that the best result was registered in the interaction between 150 mg/l nano-K and 0.75 g/l chitosan.

**Table 4.** The influence of nano-potassium and chitosan concentrations on days to flowering for initiation of inflorescence (days) and number of inflorescence per plant/month of *Strelitzia reginae*. The values are averages of the two experimental seasons of 2021 and 2022.

Days to flowering for initiation of inflorescence (days)						No. inflorescence per plant/month				
(A) Nano-K (mg/l)	(B) Chitosan (g/l)					(B) Chitosan (g/l)				
	0	0.25	0.50	0.75	M	0	0.25	0.50	0.75	M
0	45.53	43.83	42.97	41.10	43.36	0.80	1.27	1.57	1.80	1.36
100	44.47	42.77	41.40	40.23	42.22	1.10	1.50	1.80	2.53	1.73
150	43.53	42.07	40.37	39.37	41.33	1.30	2.13	2.20	2.80	2.11
200	42.83	41.27	40.53	40.40	41.26	1.10	1.60	1.90	2.60	1.80
M	44.09	42.48	41.32	40.28		1.08	1.63	1.87	2.43	
LSD 5%	A = 0.38 B = 0.32 AB = 0.67					A = 0.14 B = 0.09 AB = 0.22				

**Number of florets/ inflorescence and stalk length**

Table 5 shows that there was a significant effect of nano-K and chitosan concentrations as well as their interactions on the number of florets/inflorescence and stalk length traits. Thus, the averages of these traits significantly improved with an increase in nanoK concentrations. The plants sprayed with 150 mg/l concentration scored the highest values of number of florets/inflorescence and stalk length compared with the untreated plants, which recorded the lowest values. For chitosan concentrations, we noticed from the results in the same table that a significant effect exists among chitosan concentrations. As the concentration of chitosan increased, the number of florets/inflorescence and stalk length increased, so the 0.75 g/l gave the highest values of these traits compared with the untreated plants. Results refer that 150 mg/l nano-K and 0.75 g/l chitosan resulted in higher numbers of florets/inflorescence and stalk length traits compared with the other combinations.

**Table 5.** Influence of nano-potassium and chitosan concentrations on number of florets/inflorescence and stalk length (cm) of *Strelitzia reginae*. The values are averages of the two experimental seasons of 2021 and 2022.

No. of florets/inflorescence						Stalk length (cm)				
(A) Nano-K (mg/l)	(B) Chitosan (g/l)					(B) Chitosan (g/l)				
	0	0.25	0.50	0.75	M	0	0.25	0.50	0.75	M
0	2.20	2.50	3.13	3.30	2.78	51.33	59.83	67.70	72.10	62.74
100	2.30	2.63	3.30	3.60	2.96	53.23	68.33	73.93	75.83	67.83
150	2.50	3.50	3.97	4.50	3.62	54.37	77.77	83.33	88.77	76.06
200	2.40	3.20	3.60	3.70	3.23	56.60	74.97	80.20	83.57	73.83
M	2.35	2.96	3.50	3.78		53.88	70.23	76.29	80.07	
LSD 5%	A = 0.13 B = 0.08 AB=0.19					A = 1.07 B = 1.48 AB = 2.96				

**Stalk diameter and inflorescence weight**

As shown in Table 6, significant differences were observed between the stalk diameter and inflorescence weight averages for nano-K and chitosan spray and interactions. The plants sprayed with 150 mg/l showed the maximum average of these traits compared with the control plants. Chitosan levels have significantly influenced the stalk diameter and inflorescence weight, so the concentration of 0.75 g/l achieved the highest values of these traits as compared with untreated treatment. In return to the interactions of both study factors, the combination of 150 mg/l nano-potassium with 0.75 g/l chitosan gave the highest values of stalk diameter and inflorescence weight compared with the other combinations.

**Table 6.** Influence of nano-potassium and chitosan concentrations on stalk diameter (mm) and inflorescence weight (g) of *Strelitzia reginae*. The values are averages of the two experimental seasons of 2021 and 2022.

Stalk diameter (mm)						Inflorescence weight (g)				
(A) Nano-K (mg/l)	(B) Chitosan (g/l)					(B) Chitosan (g/l)				
	0	0.25	0.50	0.75	M	0	0.25	0.50	0.75	M
0	10.00	11.00	10.00	11.00	10.50	74.00	86.00	93.00	109.33	90.58
100	9.67	11.00	11.00	11.00	10.67	76.00	88.67	97.00	123.67	96.33
150	11.00	12.00	12.00	13.00	12.00	81.33	100.33	119.33	136.33	109.33
200	10.33	11.00	12.00	12.00	11.33	79.00	92.33	115.00	128.00	103.58
M	10.25	11.25	11.25	11.75		77.58	91.83	106.08	124.33	
LSD 5%	A = 1.20 B = 0.89 AB = 1.95					A = 1.72 B = 2.56 AB = 4.76				

**Longevity of inflorescence and flowering period**

The results in Table 7 indicate that there was a significant effect of both nano-K and chitosan and their interactions on the longevity of inflorescence and flowering period of the bird of paradise plants. It is obvious that spraying plants with 150 mg/l nano-K significantly dominated these characteristics, giving the highest values compared with the other treatments. The results also revealed that longevity of inflorescence and flowering period increased with chitosan concentrations; therefore, the 0.75 g/l achieved the highest averages of these characteristics compared to the other treatments. Regarding the interactions between the two factors, spraying plants with 150 mg/l of nano-K and 0.75 g/l chitosan gave the highest longevity of inflorescence and flowering period compared to the other combinations.

**Table 7.** Influence of nano-potassium and chitosan concentrations on longevity of inflorescence (days) and flowering period (days) of *Strelitzia reginae*. The values are averages of the two experimental seasons of 2021 and 2022.

Longevity of inflorescence (days)						Flowering period (days)				
(A) Nano-K (mg/l)	(B) Chitosan (g/l)					(B) Chitosan (g/l)				
	0	0.25	0.50	0.75	M	0	0.25	0.50	0.75	M
0	18.00	20.00	22.00	24.00	21.00	30.33	36.33	46.00	70.00	45.67
100	19.00	21.00	23.00	24.67	21.92	35.67	41.67	54.67	81.00	53.25
150	20.00	23.00	25.00	27.00	23.75	39.00	50.33	67.67	99.67	64.17
200	19.00	20.00	23.67	25.00	21.92	35.33	44.00	64.33	88.00	57.92
M	19.00	21.00	23.42	25.17		35.08	43.08	58.17	84.67	
LSD 5%	A = 1.12m B = 0.74 AB = 1.69					A = 2.55 B = 1.87 AB = 4.11				

**Chemical compositions**

**Figure 1.**
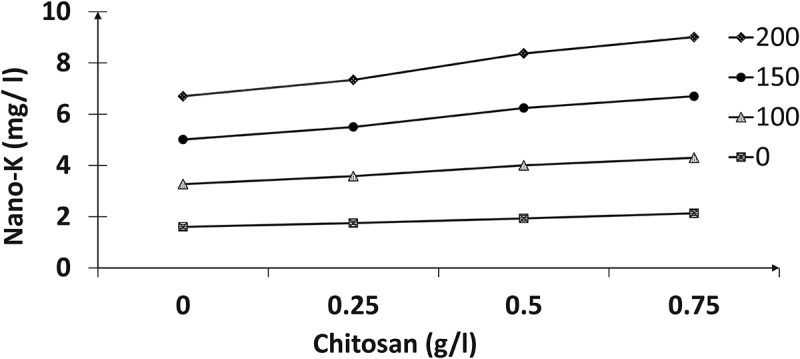
Effect of nano-potassium and chitosan on total chlorophyll (mg/gFW) of bird of paradise leaves.

**Figure 2.**
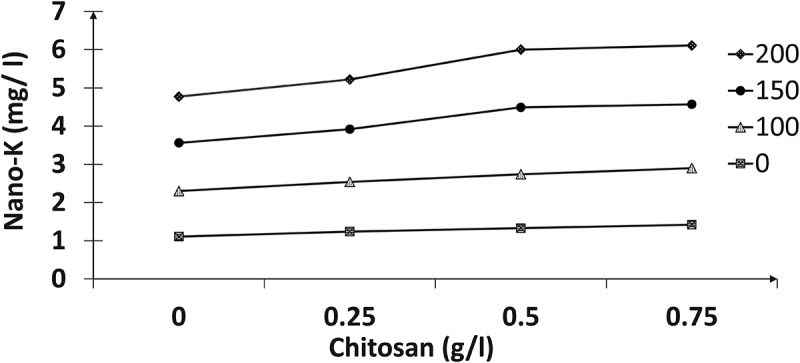
Effect of nano-potassium and chitosan on nitrogen percentage of bird of paradise leaves.

**Figure 3.**
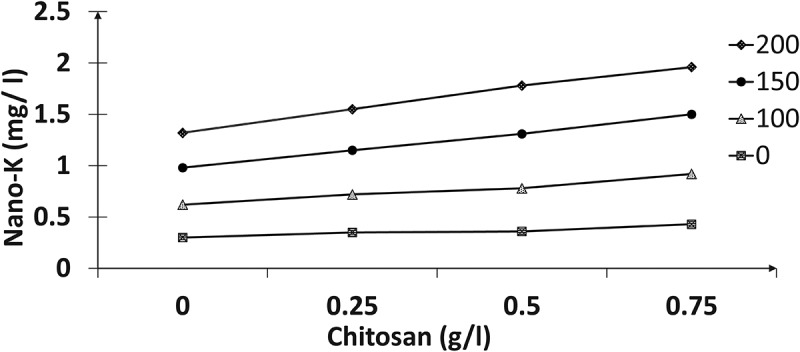
Effect of nano-potassium and chitosan on phosphorus percentage of bird of paradise leaves.

**Figure 4.**
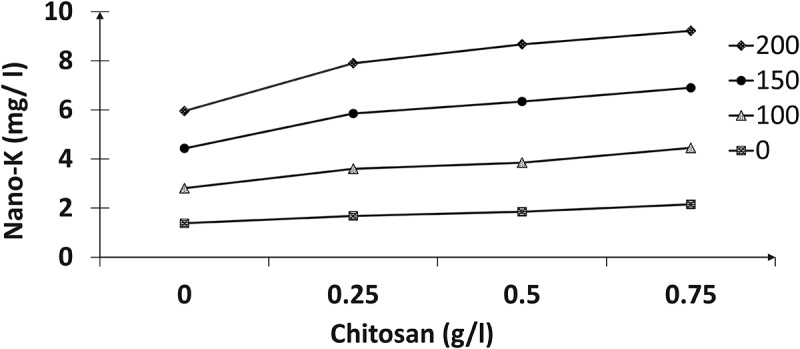
Effect of nano-potassium and chitosan on potassium percentage of bird of paradise leaves.

The results in (Figures 1–4) indicated the presence of significant effects for the tested nano-K and chitosan concentrations and their interactions on the total chlorophyll, nitrogen, phosphorus, and potassium contents of the paradise leaves. Spraying with different nano-potassium concentrations resulted in improved traits to give the highest values compared with untreated plants; higher values were recorded at 150 mg/l, followed by 200 mg/l chitosan. In addition, the results reclaimed that chemical compositions increased with increasing chitosan concentrations; therefore, the 0.75 g/l registered the highest values of these contents compared to the other treatments. Concerning the effect of interactions between the two factors, the plants sprayed with 150 mg/l of nano-K and 0.75 g/l chitosan had the highest total chlorophyll content (mg/g FW), and nitrogen, phosphorus, and potassium percentages compared to the other interactions.

## Discussion

The present study showed that applying nano-potassium and/or chitosan as sprays to birds of paradise plants led to an improvement in growth characteristics, flowering, and chemical components compared to untreated plants. In most cases, as the concentration of these stimulants increases, there is an increase in the measurements under study for plants under new soil conditions. Potassium is crucial for the formation of fat, protein, carbohydrate, and chlorophylls, and it is useful in maintaining the balance of salts and water in plant cells [27]. K activates various enzymes involved in plant growth and production of plant [28]. Moreover, the positive effect of nano-potassium concentrations, especially 150 mg/l in inducing growth and flowering, is due to nano fertilizers that can either deliver nutrients to plants or help in the absorption or transportation of available nutrients, thus leading to improved plant growth and development [29]. Mehrdad et al. [30] found that the rice plant height increased when nanoparticles were used as nano potassium. Spraying 8 ml/l nano potassium showed a higher increment in the plant height and chlorophyll content of the wheat plant. The increase in plant height of *Vicia faba* is due to the addition of potassium nutrients that stimulate cell division and cell elongation, especially in the meristematic cells in the growing tops. Furthermore, potassium functions by promoting plant growth because growth improves [31]. These results are in agreement with those reported by Parya et al. [32]; Gomaa et al. [33]; Ali et al. [34]; Al-Falahi & AbdulKafoor [35]. The enhancement in flowering characteristics of *S. reginae* plants (days to flowering, number of inflorescence/plant, number of florets/inflorescence, stalk length and diameter, inflorescence weight, longevity of inflorescence, and flowering period with potassium application) is due to its role in increasing the production of auxins that stimulate plant cell division and elongation, which increases the plant^’^s efficiency in absorbing water and nutrients [36]. Potassium also helps in the formation of chlorophyll, which increases the nutrients in the leaf, which is reflected in the flowering characteristics [37]. However, the quality of spike length and diameter in paradise birds improved with different levels of fertilizers; an increase in potassium led to an increase in stalk length [38]. K triggers the activation of enzymes and is important for ATP production, which is an important source of many chemical causes and maximum flowering characteristics. The previous results of Bhatia et al. [39] and Usha et al. [40] are in good agreement with our results.

Chitosan affects plant growth and development, and regulates metabolic and physiological processes [41,42]. In addition, chitosan encourages plants to resist various abiotic stresses such as salinity and drought [43–45]. Our results indicate that spraying birds of paradise plants with chitosan especially at 0.75 g/l had the most pronounced effect on growth and flowering traits, as well as chemical composition. Byczyńska [46] indicated that soaking *Eucomis bicolor* bulbs in 50 mg/l chitosan before planting stimulated the growth, flowering, chlorophyll content, and yield of bulbs. Similar positive effects of chitosan were observed by Salachna & Zawadzińska [47], Salachna et al. [42], and Salachna [48]. However, chitosan can be applied either as a solution for spraying and irrigating the plant or as a hydrogel for coating seeds or corms [49–51]. The results obtained by Salachna & Zawadzińska [19] have shown that chitosan can be applied as a biostimulator in the cultivation of freesia plants. A similar beneficial effect of chitosan has been observed in *Cymbidium* [52]; *Dendrobium* [53]; soy seedling growth [54]. El-Gamal & Ahmed [55] pointed out that the application of chitosan by spraying at appropriate concentrations resulted in increased plant growth parameters in terms of plant height and number of branches per coriander plant. Chitosan at higher concentrations improved vegetative growth such as plant height, leaf number per plant, leaf width, and flowering traits such as the number of florets per spike, spike length, and the fresh weight of spikes in tuberose plants [36]. In addition, spraying pineapple lily with chitosan improved inflorescence length, inflorescence width, and chlorophyll content, and began flowering earlier than untreated ones [46]. Alsanam & Salih [56] indicated that treating *Polianthes tuberosa* with 1000 mg/l chitosan resulted in a higher increment in plant growth and flowering traits. Spraying a bird of paradise with chitosan increased the leaf length, leaf width, plant height, and number of leaves per plant. These beneficial effects were reflected in the increase in leaf area and vegetative growth; in addition to the important role of chitosan as an antioxidant, vegetative growth was improved as a result of increased photosynthesis [57] and improving carbohydrates that improve growth characteristics. It increases the dissolution of sugars that act as an energy source within the plant, which improves the organic matter, and is then reflected in improving the plant fresh and dry weights [58,59].

The effect of interaction between nano-K and chitosan indicated that these interactions improved growth, flowering and chemical components compared to control plants. Indeed, the best measurements resulted from the combination between 150 mg/l nano-K with 0.75 g/l chitosan. Nano-K as a macronutrient serves the same purpose of K in protecting plants from abiotic stresses [60]. Foliar application of nano-K resulted in the increased accumulation of N, P, K and pigments, which were important indicators for plant growth and flowering [61]. On the other side, the positive effect of chitosan in enhancing the growth, flowering and availability and uptake of nutrients by regulating the cell osmotic pressure and enzyme activities [62]. Moreover, plants treated with chitosan may be less prone to stress evoked by unfavorable conditions, such as salinity [15]. Chitosan induces vital processes of the plants on every level of biological organization, from single cells and tissues, through physiological and biochemical processes, to changes on the molecular level related to expression of genes [63]. Therefore, the combination between nano-K and chitosan improved the different traits of the bird of paradise plants, especially with the application of 150 mg/l nano-K with 0.75 g/l chitosan [64].

## Conclusions

Foliar spraying of nano potassium at different concentrations, especially at 150 mg/l showed a clear enhancement in all studied traits of vegetative growth, flowering, and chemical components of the bird of paradise plants. It was proved that there is a significant response in all studied characteristics of plants with increasing chitosan concentration from 0.25 up to 0.75 g/l compared to untreated ones. The interaction between nano potassium and chitosan was significant in the studied characteristics of the bird of paradise plants, and in most cases, the best results were registered by spraying plants with 150 mg/l nano potassium and 0.75 g/l chitosan as compared to the other combinations.

## Data Availability

The datasets generated or analyzed during the current study are available upon reasonable request. In this research, we have discussed all the measurements required to be in this form. It is normal, like any study, that the required data that represent raw data are taken before it is tabulated and analyzed statistically, but we have never been asked for that raw data by any journal or publishing houses. We have never, throughout our research history, been asked for raw data for research because the final data represent all required data for the study. Hence, we are not required to provide raw data in this study.
